# Comparing maximum likelihood and Bayesian methods for fitting hidden Markov models to multi-state capture-recapture data of invasive carp in the Illinois River

**DOI:** 10.1186/s40462-023-00434-w

**Published:** 2024-01-08

**Authors:** Charles J. Labuzzetta, Alison A. Coulter, Richard A. Erickson

**Affiliations:** 1 U.S. Geological Survey, Upper Midwest Environmental Sciences Center, 2630 Fanta Reed Road, La Crosse, WI 54603 USA; 2 South Dakota State University, McFadden Biostress Laboratory 138, Box 2140B, Brookings, SD 57007 USA

**Keywords:** Bayesian methods, Hidden Markov model, Invasive species, Maximum likelihood, Multi-state capture-recapture, Population ecology, Telemetry

## Abstract

**Background:**

Hidden Markov Models (HMMs) are often used to model multi-state capture-recapture data in ecology. However, a variety of HMM modeling approaches and software exist, including both maximum likelihood and Bayesian methods. The diversity of these methods obscures the underlying HMM and can exaggerate minor differences in parameterization.

**Methods:**

In this paper, we describe a general framework for modelling multi-state capture-recapture data via HMMs using both maximum likelihood and Bayesian methods. We then apply an HMM to invasive silver carp telemetry data from the Illinois River and compare the results estimated by both methods.

**Results:**

Our analysis demonstrates disadvantages of relying on a single approach and highlights insights obtained from implementing both methods together. While both methods often struggled to converge, our results show biologically informative priors for Bayesian methods and initial values for maximum likelihood methods can guide convergence toward realistic solutions. Incorporating prior knowledge of the system can successfully constrain estimation to biologically realistic movement and detection probabilities when dealing with sparse data.

**Conclusions:**

Biologically unrealistic estimates may be a sign of poor model convergence. In contrast, consistent convergence behavior across approaches can increase the credibility of a model. Estimates of movement probabilities can strongly influence the predicted population dynamics of a system. Therefore, thoroughly assessing results from HMMs is important when evaluating potential management strategies, particularly for invasive species.

**Supplementary Information:**

The online version contains supplementary material available at 10.1186/s40462-023-00434-w.

## Background

Ecologists are embracing ever-expanding frontiers in data science to analyze data [1]. But the diversity of approaches often obscures the underlying mathematics shared among packages, programming languages, software, and terminology [2]. For example, ecologists often use multi-state capture-recapture studies to estimate movement rates among geographic locations while accounting for survival and imperfect detection [3]. But a modeler may select either a maximum-likelihood or Bayesian approach to fit these models. Additionally, a variety of packages in the R programming language or point-and-click software such as Program MARK can execute these approaches [2–4]. Despite each approach relying on HMMs as the underlying model, these various implementations often require their own specialized knowledge. A strong understanding of HMMs would benefit modelers before selecting computational methods. Such knowledge could improve one’s ability to correctly interpret the results of these analyses.

The history of capture-recapture models stems from the work of Cormack [5], Jolly [6], and Seber [7]. These studies produced the Cormack–Jolly–Seber (CJS) model, which estimates detection and survival rates through recaptures or re-encounters of individuals in repeated surveys. This model, and other capture-recapture models more generally, come in the form of a state-space model [3]. The CJS model is a state-space model with a single observable state, in which tagged individuals may be observed only in the ‘alive’ state. Multi-state models include more than one state representing observable characteristics such as geographic locations or disease status. Because these states are discrete and finite, such multi-state models can be classified as a sub-type of the state-space model: the Hidden Markov Model (HMM) [2, 8].

In this paper, we formulate HMMs with respect to both the maximum likelihood and Bayesian approaches. Our description emphasizes the HMM parameterization in both approaches and examines the effect of any special considerations that arise. We apply our study to an invasive silver carp (*Hypophthalmichthys molitrix*) telemetry dataset from the Illinois River. Previously, Coulter et al. [9] presented an analysis of these data using Program MARK. Although many studies use these models and methods to analyze capture-recapture data [9–12], few compare implementations of both the maximum likelihood and Bayesian approaches on the same dataset (as notable exceptions, refer to Kéry and Royle [3]; Kéry and Schaub [13]).

Understanding the spatial dynamics of invasive species is a vital step in managing and limiting their spread [14]. Advances in the development of widespread and low-cost sensor arrays have greatly improved the feasibility of monitoring spatial dynamics of aquatic invasive species, such as silver carp [1]. For example, several institutions monitor and prevent the movement of invasive bigheaded carps (Genus: *Hypophthalmichthys*) from the Illinois River into the Laurentian Great Lakes [9, 15]. Collaborators are particularly interested in determining where innovations such as movement deterrents, targeted removal, and further monitoring would be useful to better manage and understand the effects of invasive carp in the Illinois River system [16].

**Fig. 1:**
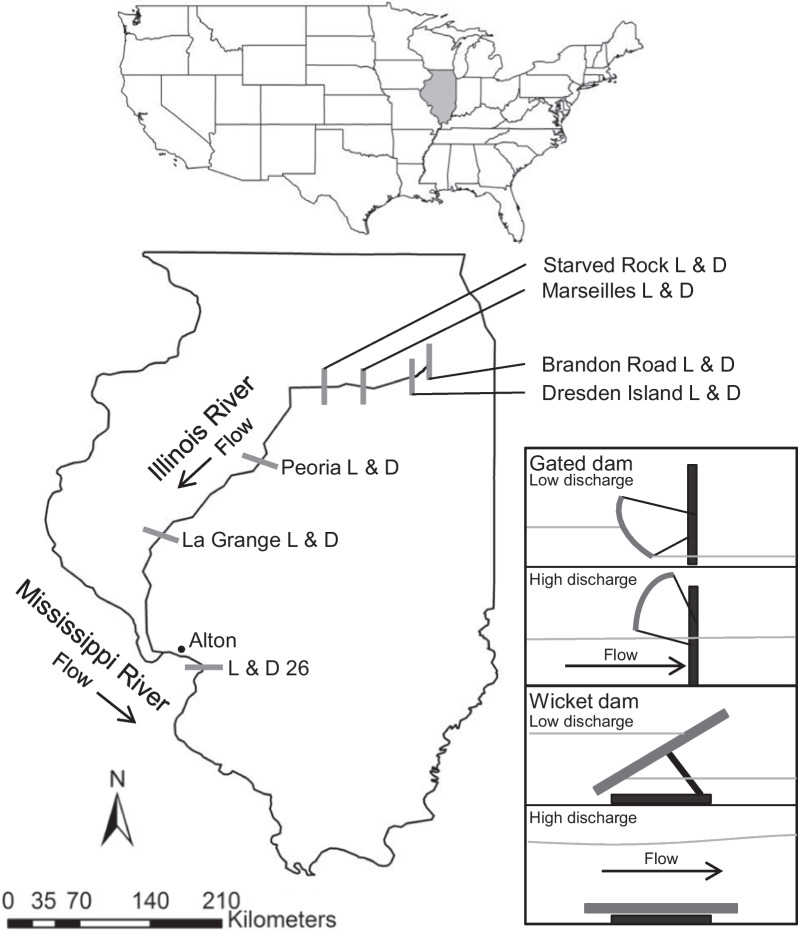
A geographic description of the Illinois River and the locations of the Lock & Dams (L & D) that separate the pools. The Illinois River flows downstream toward the Mississippi River from the Dresden Island Pool, which starts at Dresden Island L & D. Upstream the Brandon Road L & D and several other structures act as additional barriers between the Dresden Island Pool and Lake Michigan. Each pool downstream is named after the L & D where it starts, except for the most downstream pool, Alton, which is named after the town near the confluence of the Illinois and Mississippi Rivers. The Dresden Island, Marseilles, and Starved Rock dams are each gated dams, whereas Peoria, La Grange, and L & D 26 are wicket dams. Figure adapted from Coulter et al. [9]

Many studies use capture-recapture models to estimate the spatial dynamics of invasive species [9, 12, 14, 17]. HMMs can model the movement of aquatic species in rivers that are segmented into reaches or pools by dams. These models estimate the probability an individual moves from one pool to another over time [9, 12]. The existing dams in the Illinois River limit movement but are not impermeable barriers to invasive carp and other fish [18, 19]. Thus, estimating movement probabilities among pools in the Illinois River may help to determine locations where the installation of deterrents and targeted removal may be most effective [9, 18, 20, 21]. Figure 1 shows the dams in the Illinois River, and Section “Data” provides further description of this system.

In this paper, we estimate parameters similar to an HMM described in Coulter et al. [9]. Our primary objective is to compare parameter estimates among three methods for fitting this model (1) maximum likelihood in R; (2) Bayesian Markov-chain Monte Carlo (MCMC) estimates via an R Interface to CmdStan; and (3) the Coulter et al. [9] results using Program Mark. Our goal is to show how estimated population dynamics can vary across approaches and how this variability could influence ecological management decisions depending on the parameter estimates produced by each method. Through this exploration of various methods for fitting HMMs we discuss how to overcome computational challenges that may be inherent in HMMs due to weak parameter identifiability given sparse data. Specifically, we explain that under some parameterizations, both the maximum likelihood and Bayesian MCMC approaches produced poor convergence, so we used informative priors and initial values to guide convergence toward biologically realistic solutions. This paper provides a foundation for other scientists looking to study their own telemetry data with HMMs and provides awareness of the particular challenges that may arise in these analyses.

## Methods

### Hidden Markov models

HMMs mathematically represent sequential observations. Ecologists often apply HMMs to repeated observations of individuals over time. Various methods for tracking individuals exist including tagging, marking, or recording unique patterning to render each individual uniquely identifiable. For example, acoustic receivers can track the presence of tagged individuals across geographic states over time [22].

The mathematics of HMMs represent such data well due to several ecological phenomena: (1) an individual might die but its death is not directly observed; and (2) an individual might survive but remains undetected for one or more survey periods. HMMs account for these phenomena through two processes: the state process and the observation process. The state process is a hidden (i.e., latent) variable. The state process represents the true but generally unknown states of the individuals in the sequence, and the observation process records the state process in an imperfect manner. For example, an individual might be present at location ‘A’ during a survey occasion, but the researcher or receiver fails to detect this individual. In this case, the true state of the individual is ‘A,’ but the true state is unknown and recorded as a non-detection in the observation process. This is an example of imperfect detection known as a false-negative, a common event in ecological studies. False-positives, where an individual is incorrectly recorded as being in a state different than its true state, are another type of imperfect detection. The HMM plays a fundamental role in most multi-state capture-recapture studies due to its ability to model such ecological phenomena.

To represent such datasets, we now formulate the HMM based on the descriptions by Kéry and Royle [3] and Kéry & Schaub [13]. Consider a group of $N$ tagged individuals that move among a set of $M$ discrete geographic locations or areas. Over a set of $T$ occasions, each individual $i \in 1, 2, \ldots , N$ has a state process ${\textbf{S}}_i = S_{i,1}, S_{i,2}, \dots , S_{i,T}$ that represents the true but unknown state (i.e., location) of the individual at each occasion. $S_{i,t}$ may be any geographic state $1, 2, \ldots ,M$, but may also be state $M+1$, which occurs when the individual is no longer alive. At each occasion $t$, we record an observation $Y_{i,t}$, which may be equal to any state $1, 2, \ldots ,M$, or a non-detection event, which is recorded as $M + 1$. Even though $Y_{i,t}$ may be equal to $M+1$ this is not necessarily equivalent to $S_{i,t} = M+1$. In the observation process, a non-detection $Y_{i,t} = M+1$ may occur because the individual has not survived ($S_{i,t}=M+1$) or it simply was not detected despite the true state $S_{i,t}$ being one of $1, 2, \ldots , M$.

The HMM is also built on two additional assumptions: (1) the probability of the state process being in $S_{i,t}$ depends only on the previous state $S_{i,t-1}$; and (2) the probability of observing $Y_{i,t}$ at any occasion depends only on the state process value $S_{i,t}$. Formally, the first assumption is known as the Markov Property where $\text {Pr}(S_{i,t} | S_{i,t-1}, S_{i,t-2},\ldots ) = \text {Pr}(S_{i,t} | S_{i,t-1})$. To specify these probabilities, consider a parameterization of the HMM with the following components.

The initial distribution is a vector that describes the probability of the first observation belonging to each state, given below in Eq. 1.

1 $$\begin{aligned} \varvec{\delta }_i = (\text {Pr}(S_{i,1} = 1), \dots , \text {Pr}(S_{i,1} = M), \text {Pr}(S_{i,1} = M+1)) \end{aligned}$$

In a multi-state capture-recapture study, the initial distribution is considered known when an individual is captured, tagged, and released in a specific state, because we can set the probability of the known state to 1 and all others to 0.

Additionally, we consider the state transition probability matrix that specifies the probability of moving from state $j$ at time $t-1$ to state $k$ at time $t$ as $\psi _{j,k} = \text {Pr}(S_{i,t} = k | S_{i,t-1} = j)$. We require $\sum _{k=1}^{M+1} \psi _{j,k} = 1$. The state $M+1$ occurs when the individual is no longer alive; therefore, movement to other states must be set to 0 and $\text {Pr}(S_{i,t} = M+1 | S_{i,t-1} = M+1) = 1$, because these individuals can no longer move to another state (refer to Eq. 2). We consider $\psi _{j,k}$ for $j, k \in 1, 2, \ldots ,M$ to represent movement and the parameter $\phi _j$ to represent survival in state $j$ before movement to state $k$.

2 $$\begin{aligned} \Psi = \mathop {\left[ {\begin{array}{*{20}c} \phi _1\psi _{1,1} &{} \cdots &{} \phi _1\psi _{1,M} &{} 1-\phi _1 \\ \vdots &{}\quad \ddots &{}\quad \vdots &{}\quad \vdots \\ \phi _M\psi _{M,1} &{}\quad \cdots &{}\quad \phi _M\psi _{M,M} &{}\quad 1-\phi _M \\ 0 &{}\quad \cdots &{}\quad 0 &{}\quad 1 \\ \end{array} } \right] }\limits ^{{\begin{array}{*{20}l} S_{i,t-1} = 1 &{} \dots &{} S_{i,t-1} = M &{} S_{i,t-1} = M+1 \\ \end{array} }}\begin{array}{cccc} S_{i,t}=1 \\ \vdots \\ S_{i,t}=M \\ S_{i,t}=M+1 \\ \end{array} \end{aligned}$$

**Fig. 2:**
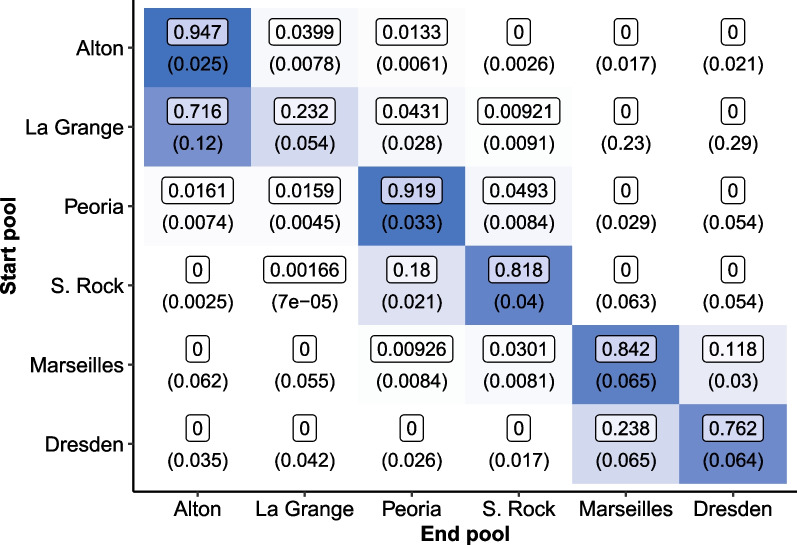
Estimates for the monthly transition probabilities of silver carp among pools in the Illinois River via maximum likelihood estimation. Movement is represented from row to column. Standard errors are reported in parentheses. Additionally, the color intensity of each cell indicates the magnitude of the estimated movement probability

**Fig. 3:**
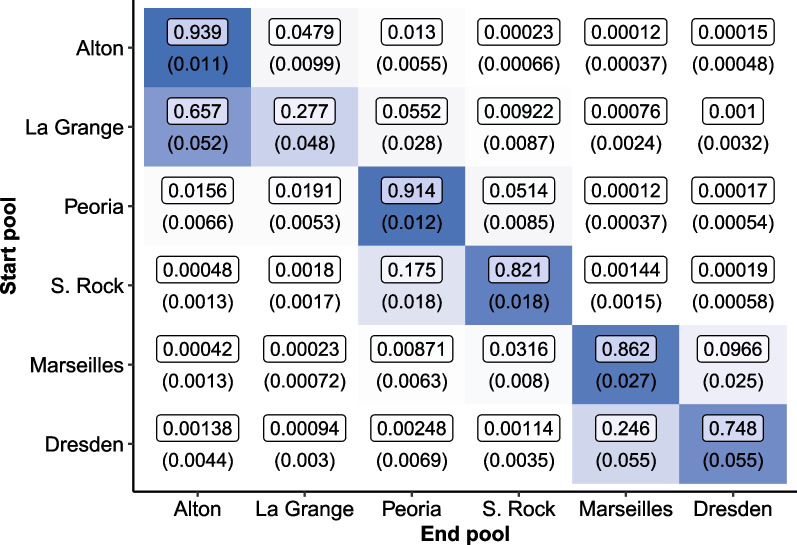
Estimates for the monthly transition probabilities of silver carp among pools in the Illinois River via Bayesian Markov-chain Monte Carlo estimation. Movement is represented from row to column. Standard errors are reported in parentheses. Additionally, the color intensity of each cell indicates the magnitude of the estimated movement probability

We can also consider a matrix of the transition probabilities $\psi _{j,k}$ for $j, k \in 1, 2, \ldots ,M$ alone to represent movement rates without accounting for survival as shown in Figs. 2, 3 and 5.

Finally, consider the observation probability matrix $\text {P}(Y_{i,t})$ in Eq. 3. We parameterize the observation probability matrix $\text {P}(Y_{i,t})$ as a function of the observed value $Y_{i,t}$ at each survey occasion and the function that determines the detection probability parameter as function of time $p_{k}(t)$ for $k \in 1, 2, \ldots ,M$.3Note that all of the survival, movement, and detection parameters may be estimated as a function of time or considered constant across time depending on the goals or context of the model. For example, in our application, we consider the detection parameters $\rho _{k,s}$ to be determined by a function of time $p_k(t)$ such that $s \in 1, 2, \ldots ,4$ based on four seasons as described in Eq. 4.

4 $$\begin{aligned} p_{k}(t)= {\left\{ \begin{array}{ll} \rho _{k,1} &{} \text {if } \text {month}(t) \in \{\text {Dec},\text {Jan},\text {Feb}\}\\ \rho _{k,2} &{} \text {if } \text {month}(t) \in \{\text {Mar},\text {Apr},\text {May}\}\\ \rho _{k,3} &{} \text {if } \text {month}(t) \in \{\text {Jun},\text {Jul},\text {Aug}\}\\ \rho _{k,4} &{} \text {if } \text {month}(t) \in \{\text {Sep},\text {Oct},\text {Nov}\} \end{array}\right. } \end{aligned}$$

These matrices are multiplied together via the forward algorithm to form the likelihood (Eq. 5) as a function of the unknown parameters $\varvec{\theta } = (\varvec{\phi }, \varvec{\psi }, \varvec{\rho })$ and the observation process ${\textbf{Y}}_i = Y_{i,1}, \ldots ,Y_{i,T}$ of each individual $i \in 1, 2, \dots ,N$.

5 $$\begin{aligned} {\mathcal {L}}(\varvec{\theta }; {\textbf{Y}}_i) = \prod _{i = 1}^N \varvec{\delta }_i \Psi \text {P}(Y_{i,2}) \Psi \text {P}(Y_{i,3}) \dots \Psi \text {P}(Y_{i,T}) {\textbf{1}} \end{aligned}$$

There are two common approaches to estimating the parameter values $\varvec{\theta }$ and fitting an HMM: the classical, maximum likelihood approach and the Bayesian approach via MCMC. In both cases, the likelihood function plays a fundamental role in estimating the parameters $\varvec{\theta }$, which describe the rates of survival, movement, and detection across the sampled individuals in the system.

### Maximum likelihood approach

The maximum likelihood approach comes from the classical or “frequentist” perspective of statistics. We estimate the parameters by finding the values $\hat{\varvec{\theta }}$ that maximize ${\mathcal {L}}(\varvec{\theta }; \mathbf {Y_i})$. The function ${\mathcal {L}}(\varvec{\theta }; \mathbf {Y_i})$ is an expression of the HMM’s likelihood in the form of the forward algorithm. We can use both numerical and theoretical methods to solve for maximum likelihood estimates, but complex likelihood functions often prevent deriving these estimates theoretically. Numerical algorithms can optimize the parameters of a likelihood function to find the maximum likelihood estimate even if the partial derivatives of the likelihood function are mathematically intractable.

### Bayesian MCMC approach

Bayesian statistics describe the posterior distribution. The likelihood ${\mathcal {L}}(\varvec{\theta }; \mathbf {Y_i})$ is incorporated into the calculation of the posterior (Eq. 6), but this distribution is interpreted differently than the likelihood.

6 $$\begin{aligned} p(\varvec{\theta } | {\textbf{Y}}_i) \propto {\mathcal {L}}(\varvec{\theta }; {\textbf{Y}}_i) p(\varvec{\theta }) \end{aligned}$$

The posterior $p(\varvec{\theta } | {\textbf{Y}}_i)$ represents the probability of the parameters $\varvec{\theta }$ given the observed data ${\textbf{Y}}_i$. The posterior is numerically proportional to the product of the likelihood ${\mathcal {L}}(\varvec{\theta }; {\textbf{Y}}_i)$ and the prior distribution $p(\varvec{\theta })$. The prior distribution is a joint distribution on the parameters $\varvec{\theta }$, which describes “prior knowledge” about the distributions of these parameter values. The posterior distribution of each parameter is influenced by prior selection, so modelers often desire relatively uninformative priors [23]. In some cases, however, informative priors may be selected to help guide inference for computational or statistical purposes [23–25]. We discuss the selection of priors for our model in Sect. “Bayesian Markov-chain Monte Carlo”.

The theoretical form of the posterior distribution for our model is mathematically intractable. Therefore, we use a computational technique to approximate the posterior distribution and estimate the parameters $\varvec{\theta }$. We use MCMC to simulate a large sample of observations drawn randomly from the posterior. This distribution can then be used to calculate the mean value and other statistics for each parameter in $\varvec{\theta }$.

### Data

In this paper, we reanalyzed acoustic telemetry data of invasive silver carp in the Illinois River previously described in Coulter et al.  [9]. These tagged individuals pass by receivers in the Illinois River and can be uniquely identified, making it possible to track movement of these fish among discrete sections of the river over time. The Illinois River is divided into pools, including Dresden Island, Marseilles, Starved Rock, Peoria, La Grange, and Alton by dams that preserve the navigability of the river for barges and other large vessels. The uppermost pool in our study is Dresden Island, which is 23 river kilometers (rkm). This pool flows into Marseilles (39 rkm) and subsequently Starved Rock (26 rkm). The lower pools follow, including Peoria (118 rkm), La Grange (125 rkm), and Alton (129rkm), which starts at L & D 26 on the Mississippi River [26]. Refer to Fig. 1 for a map of the locations of these structures. The Dresden Island Pool is considered the current invasion front for bigheaded carp [16], where invasive carp are beginning to invade but have not yet established large populations. Monitoring has shown the upper pools, Marseilles and Dresden Island, have notably lower bigheaded carp abundance than the lower pools [27].

A stationary acoustic telemetry array tracked bigheaded carp movement from the Illinois-Mississippi River confluence to the Dresden Island Pool between March 2012 and August 2015 [9]. Vemco receivers (VR2, VR2W, VR2Tx; Vemco, Bedford, Nova Scotia, Canada) were placed in each pool in locations designed to detect movement among pools and through L & D structures [9]. Gated dams separate the upper pools whereas wicket dams separate the lower pools, which results in lower movement rates among the upper pools except during open water conditions [26]. Some receivers were also placed in lateral habitats to the main channels to increase the probability of detection in areas that fish congregate. Program MARK was used in Coulter et al. [9] to parameterize multi-state capture-recapture models for these telemetry data from the Illinois River.

In this paper, we reanalyzed the raw silver carp dataset from Coulter et al. [9]. We repeated many of the same processing and formatting steps, but we implemented the process programmatically in R to ensure reproducibility. These steps generated capture histories for $N = 525$ silver carp. Each capture history had monthly redetection periods between March 2012 and August 2015 with variable initial capture-tag-release dates. We considered only the final detection of each fish per month to represent observations. Non-detection values were included when a fish was not detected during the entire month. The final capture histories generated via this programmatic approach differed slightly from the data used in Coulter et al.  [9]. We recovered histories for eight additional individuals and included several months of data that were left out of the original analysis. Although the datasets are not identical between this paper and the previous work, we expect the discrepancies to have only a minimal effect on the overall analysis and comparison.

Table 1 describes the number of recorded transitions between each pool after removing all non-detections. Additionally, Table 2 provides the total number of tagged fish starting in each pool. From these values, we hypothesize the transition and detection rates among pools should vary strongly. The maximum likelihood and MCMC approaches will each provide an interpretation of the movement and detection dynamics that underlie these raw data, but it will also be important to consider whether the estimates describe our prior biological knowledge of the system appropriately.

**Table 1:** After removing all non-detection events from the capture histories, this table provides the frequency of observed silver carp transitions between subsequent detections

	Alton	La Grange	Peoria	S. Rock	Marseilles	Dresden
Alton	78	23	4	1	0	0
La Grange	42	34	4	2	0	0
Peoria	5	13	93	4	0	0
S. Rock	1	3	39	578	0	0
Marseilles	0	0	2	15	381	5
Dresden	0	0	0	0	4	5

Transitions occur from rows to columns. For example, 42 silver carp were detected moving from La Grange to Alton, but there may have been zero, one, or more non-detection events that occured between the detections in these pools

**Table 2:** The frequency of capture histories of silver carp (n) that begin in each of the six pools of the Illinois River

Pool	n
Alton	72
La Grange	54
Peoria	111
Starved rock	151
Marseilles	133
Dresden Island	4

### Model parameterization

Consider the six pools in the Illinois River: $S_1 = \text {Alton}$, $S_2 = \text {La Grange}$, $S_3 = \text {Peoria}$, $S_4 = \text {Marseilles}$, $S_5 = \text {Starved Rock}$, $S_6 = \text {Dresden Island}$, and $S_7$ signifies the individual has died, permanently emigrated, or the battery in the tag has died. Therefore, the parameter $\phi$ does not represent true survival, rather ‘apparent’ survival (as it is in most capture-recapture models where movement out of the population cannot be distinguished from mortality) because $\phi$ may represent permanent emigration or other situations where the individual can no longer be detected in the system for reasons other than death. Additionally, a value $Y_t = 7$ indicates a non-detection for month $t$. The initial state that each fish was captured, tagged, and released was considered known and represented as such in $\varvec{\delta }_i$. In Coulter et al.  [9], battery failure was considered an additional state, but we found it unnecessary to separate a battery failure state from our apparent survival parameter $\phi$ for the purposes of this analysis, which focuses on movement and detection probabilities. In this case, the apparent survival parameter can be interpreted as the rate fish drop out of the study due to either battery failure, emigration, or death. All other parameters have a directly comparable counterpart between the HMM described in this paper and those previously described in Coulter et al. [9].

Based on the top model fit of the silver carp model in Coulter et al. [9], we parameterized the model in this paper with a constant apparent survival probability $\phi$ across time and all pools, separate transition probabilities among each pool but constant across time, and detection probabilities varying by pool and season. A four season structure was implemented by grouping observations in months Dec–Feb, Mar–May, Jun–Aug, and Sep–Nov. This parameterization is given by the function for the detection probability parameters in Eq. 4.

### Estimation methods

#### Maximum likelihood

In this paper, we numerically optimized the likelihood function ${\mathcal {L}}(\varvec{\theta }; \mathbf {Y_i})$ with the R programming language (v4.2.1) using the optim function [28]. Several modifications to the original likelihood function (Eq. 5) were necessary to estimate the parameters in our model, however. Because each parameter is a probability, we restricted each optimization range to the values in $[0,1]$ via box constraints. Box constraints enforce an upper and lower bound on each parameter using the ‘L-BFGS-B’ algorithm [29]. Additionally, the sum of the probabilities from one pool to all others must equal $\sum _{k=1}^{M} \psi _{j,k} = 1$. In the maximum likelihood model, we enforced this constraint by setting each movement parameter where $j = k$ to be equal to $1 - \sum _{j \ne k} \psi _{j,k}$ for each $j$ and all $k \in 1, 2, \ldots ,M$. For example, we let $\psi _{1,1} = 1 - \psi _{1,2} - \psi _{1,3} - \psi _{1,4} - \psi _{1,5} - \psi _{1,6}$.

Alternatively, we could have used multinomial-logit link functions to transform the range of the parameters between $-\infty$ and $\infty$, but we found this had little effect on the final estimates and made calculating the variance of the estimates considerably more complex. Optimization in R is often performed as a minimization procedure rather than maximization. Therefore, we multiplied the likelihood by $-1$ and used a log-transform for computational stability. Using optim with the ‘L-BFGS-B’ method and box constraints, we minimized the negative log likelihood $-\log ({\mathcal {L}}(\varvec{\theta }; {\textbf{Y}}_i))$ to find the parameter values that optimized this function, which produced the maximum likelihood estimates $\hat{\varvec{\theta }}$.

The variance of the parameter estimates under the maximum likelihood approach may be estimated as the inverse of the Hessian estimator. However, computationally solving for the inverse of this matrix may produce a matrix with negative quantities, which cannot be used to represent a covariance matrix. We used a method described by Gill & King [30] to calculate a pseudo-variance matrix due to negative covariances approximated by the inverse Hessian matrix. Following this guidance, we calculated the nearest positive definite matrix to the inverse Hessian, which allowed us to approximate the standard error of all estimated parameters. Additionally, for the constrained movement parameters $\psi _{j,k}$ where $j = k$ as described above, we used the delta method to calculate their standard error as a function of estimated parameters. Of note, the standard errors for the movement probabilities where no movement between pools was observed would be expected to have poor estimability due to sparse data.

#### Bayesian Markov-chain Monte Carlo

In this paper, we performed Bayesian MCMC estimation using the cmdstanr package (v2.30.1) in R (v4.2.1) [28, 31]. We chose CmdStan as our MCMC sampling software due to its computational efficiency. Recently, Yackulic et al. [32] showed marginalized sampling methods can greatly improve sampling efficiency, especially when implemented with Stan based samplers compared to JAGS and BUGS. Sample code from Yackulic et al. [32] and Kéry & Schaub [33] helped us implement this approach.

As described in Sect. “Bayesian MCMC approach”, Bayesian analyses require the selection of priors for each parameter. The priors can describe the distribution of the parameters in a manner ranging from uninformative to strongly informative. Uninformative priors have minimal influence on the posterior distribution. In contrast, we can select an informative prior to influence the posterior distribution if there is pre-existing knowledge of the probability distribution of the parameters or the MCMC algorithm does not converge under weaker priors [23–25]. In this analysis, we selected priors and constraints that could become increasingly informative to ensure convergence to biologically realistic solutions.

We assigned Beta priors to the detection and survival parameters, $\phi$ and $\varvec{\rho }$, initially with $\alpha = 1, \beta = 1$ (corresponding to a uniform distribution). To constrain the transition probabilities to sum to one, we considered a ratio of a Gamma prior to the sum of $\text {Gamma}(\sigma _{j,k},\theta _{j,k})$ priors for each movement probability. For example, $\psi _{1,1} = \gamma _{1,1}/\sum _{k=1}^6 \gamma _{1,k}$ and each $\gamma _{1,k} \sim \text {Gamma}(\sigma _{1,k},\theta _{1,k})$. Under these priors, one Markov chain did not behave like the others and produced unique estimates for several movement and detection probability parameters. We discuss the implication of this finding in Section “Discussion”.

To encourage convergence among chains via our Bayesian MCMC approach, it was necessary to select more informative priors [23]. We decided to choose biologically informative priors for the movement and detection parameters to guide convergence toward a biologically realistic solution. In the Illinois River system, each pool connects only to the pools immediately adjacent in a linear fashion. If a fish moves from one pool to another that is several pools upstream, this fish would have to move through several L & D structures. Given these barriers to fish movement, we expect most fish to stay in a single pool during a one-month time step or move to an adjacent pool with lower probability. We expect it is even less likely a fish will move through two or more pools in a single month.

Therefore, we selected priors and initial values for the movement probabilities to reflect the generally higher probability an individual would remain in the same pool from one month to the next (~ 63%), with a moderate probability of entering an adjacent pool (~ 16%), and a low probability of entering any non-adjacent pool (~ 1.5%). These percentages are a result of the values selected for the parameters of the Gamma prior distributions on the movement probabilities. We selected these values by incrementally decreasing the probability an individual would move between non-adjacent pools until convergence among all chains was observed.

Selecting the priors in this manner allowed us to guide convergence based on our prior knowledge of the biological system using increasingly informative priors [23–25]. In essence, these assumptions are similar to an adjacency matrix constraint but our structure includes the possibility that a fish may travel across multiple pools in a single month, given some individuals are known to travel a long distance [17, 34]. Refer to Section “Biologically informative priors and intial values guide convergence toward realistic solutions” for a comparison between the movement probabilities estimated under non-informative priors versus the informative priors described above, which produce parameter estimates that are biologically realistic across all chains compared to those under the non-informative priors.

Additionally, we assumed a $\text {Beta}(2, 1)$ prior for each detection and survival probability to specify a relatively weak belief that survival and detection probabilities should generally be higher rather than lower. For full specification of the priors and initial values, refer to the Additional file 1: Section 9.1.

## Results

Our estimation of the seasonal detection probabilities were generally similar to those reported in Coulter et al. [9] for both the maximum likelihood and Bayesian approaches. Detection probabilities were consistently among the lowest in Dresden Island Pool and high in both Starved Rock and Marseilles. On the other hand, our models reported higher detection probabilities for La Grange Pool rather than Alton, and our estimated detection probabilities were slightly lower for Peoria Pool than those reported in Coulter et al. [9]. Refer to the Additional file 1: Section 9.2 for detailed summaries of all parameter estimates. Similar to Coulter et al. [9], throughout the rest of this analysis we described our results focusing on the movement probabilities.

### Maximum likelihood

The maximum likelihood approach generated estimates in under an hour, but successful convergence was dependent on algorithm selection. Figure 2 reports estimated maximum likelihood transition probabilities between each pool and the standard error of each estimate in parentheses. Successful convergence was reported by the ‘L-BFGS-B’ algorithm within 1000 iterations. Convergence is achieved when the magnitude of the largest element in the projected gradient is less the pre-determined tolerance level [29]. Investigating whether the algorithm converges to the same parameter estimates under a variety of initial values is important to check for convergence to the global solution rather than a local optimum.

### Bayesian Markov-chain Monte Carlo

Each of the 4 chains for the final Bayesian model ran for 5000 warm-up iterations and 10,000 sampling iterations. These samples create an empirical posterior distribution for each parameter—a process that required several hours. Figure 3 reports the transition probability estimates between each pool and a standard error for each estimate in parentheses.

**Table 3:** The total computation time and computing resources used to produce the final parameter estimates for the maximum likelihood and Bayesian MCMC approaches

Method	Computation time	Parallelized	Resources
Maximum likelihood	34 min	No	1 CPU
Bayesian MCMC	7 h 16 min	Yes	4 CPUs

The Bayesian MCMC approach required more time and resources, but equivalent parameter estimates with lower but still acceptable effective sample sizes could likely have been achieved with a reduced number of total sampling iterations to lower the run time

Convergence of the Bayesian MCMC estimation can be assessed via Gelman-Rubin (${\hat{R}}$) and Effective Sample Size (ESS) statistics across all parameters. Refer to the Stan User Manual [35] for a description of how ${\hat{R}}$ and ESS are calculated and Vehtari et al. [36] for additional discussion on these metrics. We report these values in the Additional file 1: Section 9.2. Under the final model (including informative priors) all ${\hat{R}}$ values were less than 1.001 and the minimum ESS was 12349. Recommended cutoffs for these statistics range between less than 1.01–1.1 for the Gelman-Rubin statistic and at least 100 to several thousand bulk and tail ESS per chain [35, 37]. The convergence statistics for our final model using informative priors outperformed all of these recommendations, but the model using uninformative priors did not achieve convergence; many ${\hat{R}}$ values were greater than 1.1 (at maximum 1.52) and many ESS values were less than 100.

Our final Bayesian MCMC model was likely run for more iterations than needed to reach convergence. This should be taken into account when comparing the computational burden of the Bayesian MCMC method to the maximum likelihood approach. Equivalent parameter estimates with lower but still acceptable ESS could likely have been achieved with a reduced number of total sampling iterations to lower the run time. With this in mind, consider the total run time for each method reported in Table 3. Despite these caveats, it is reasonable to conclude the Bayesian MCMC approach required more time and resources than the maximum likelihood approach.

## Discussion

### Achieving convergence is difficult with both methods

The model we have described has many parameters. Each pool has a transition parameter between itself and all other pools, and each pool has a detection parameter for each season. Additionally, a constant parameter estimates apparent survival. Even the most constrained version of our model includes 55 variables to optimize.

The movement matrix could be constrained to an adjacency matrix to improve the estimability of the movement parameters. Similarly, we could set selected movement probabilities equal to 0 if there is no expected movement between distant pools within a month given the raw data. Evidence indicates, however, that bigheaded carp may migrate over long distances in short time periods [17, 34]. Additionally, managers are particularly interested in estimating movement probabilities to and from the Dresden Island Pool, which is considered the invasion front for bigheaded carp in the Illinois River. Therefore, constraining the model to reduce the number of movement parameters would simplify the biological system to an unreasonable degree.

After minimizing the parameter space as much as possible, there are 55 parameters to optimize in the maximum likelihood model. We investigated a variety of optimization approaches including various algorithms (for example: ‘Nelder-Mead’, ‘BFGS’, ‘L-BFGS-B’, ‘SANN’), link functions (for example: identity versus multinomial-logit), and various initial value schemes. Each method had its own challenges and trade-offs. Several optimization approaches converged in relatively few iterations, but calculating estimates of standard error was often notably more difficult or numerically challenging due to errors when inverting the Hessian matrix. For this reason, we selected a standard algorithm with box-constraints to estimate the parameters as probabilities directly, without a link function. The ‘L-BFGS-B’ algorithm required nearly 1000 iterations to reach relative convergence, but this approach is still notably more efficient than sampling all parameters via Bayesian MCMC estimation.

Due to its dependence on the data alone, the maximum likelihood approach optimizes toward a movement probability equal to zero for movement parameters between pools where no movement was observed. Given the lack of observations, the estimability of these parameters is poor, which results in high variability. On the other hand, the Bayesian approach estimates small but non-zero probabilities of movement between distant pools with no observed movement due to dependence on prior distributions and stochastic sampling via MCMC. That being said, practitioners would ideally limit the strength of such priors with care to prevent the posterior distribution from being inadvertently biased.

Running a Bayesian MCMC estimation for many iterations can help ensure convergence has been achieved. While fitting our Bayesian HMM via MCMC, we noticed the efficiency varied greatly depending on the implementation and sampling software. We initially programmed our sampling algorithm in JAGS, but found that sampling for even a few-hundred iterations required several hours. Following the guidance of Yackulic et al. [32], we designed a marginalized sampling method in cmdstanr that greatly improved the efficiency of the MCMC sampling [31]. With this approach we could test the convergence of various parameterizations over several thousand iterations within an hour. However, we found evidence that informative priors were necessary to produce convergence across all chains to a single solution. Selecting biologically informative priors can help guide convergence toward realistic solutions [23, 25], which we discuss in the following section.

**Fig. 4:**
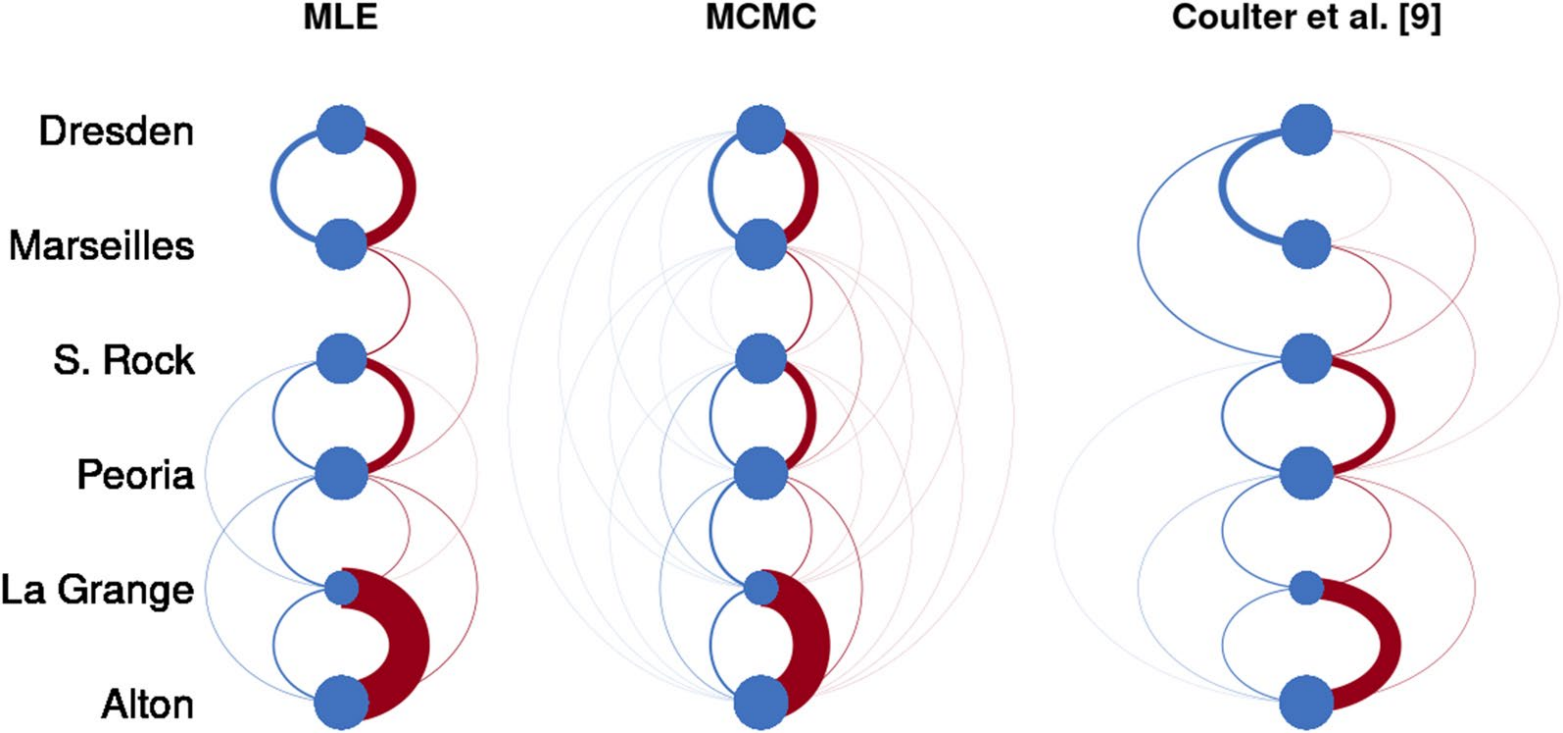
A graphical depiction of movement probabilities via an arc diagram comparing results from maximum likelihood (MLE) and Bayesian Markov-chain Monte Carlo (MCMC) with informative priors to the results from Coulter et al. [9]. Thick lines represent stronger movement probability. Lines in blue (left) represent upstream movement between pools, and lines in red (right) represent downstream movement between pools

### Biologically informative priors and intial values guide convergence toward realistic solutions

HMM states with few observations may result in weak identifability among the associated movement and detection probability parameters in the model. Identifiability is the ability to achieve unique parameter estimates given the nature of the data, model, and objective function [38]. In our application, multiple parameter estimates could empirically explain the sparse observations in some states. For example, few observations may indicate the detection probability for a state is relatively low but movement to this state could still occur at a high rate without detection. On the other hand, the state could have a relatively high probability of detection but few individuals move into this state.

From the data alone, it is difficult to determine the reality of each situation. To improve the identifiability of the parameters under sparse data, the model can be constrained either through assumptions under the maximum likelihood framework or by using informative prior distributions in the Bayesian context. Maximum likelihood inference is a function of the data alone, but model constraints and initial values can be chosen to guide parameter estimation. In contrast, the Bayesian approach requires priors, which we can specify in either an uninformative manner or to reflect prior knowledge in the probability distributions of the parameters [23–25]. The Bayesian approach gives us slightly more flexibility to guide convergence based on prior knowledge of the biological system, but implementing these assumptions without careful thought may inadvertently bias the posterior.

**Fig. 5:**
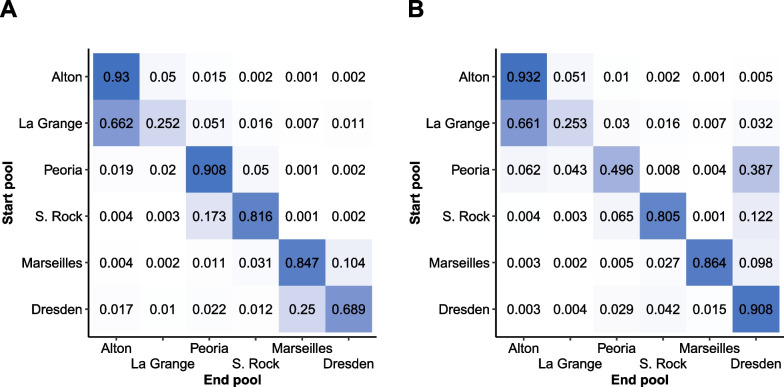
A comparison of movement probability estimates between several Markov-chain Monte Carlo chains that converged to different solutions. The color intensity of each cell indicates the magnitude of the estimated movement probability. Chains 1–3 converged to a solution that is biologically realistic (Scenario **A**); strong movement occurs only between adjacent pools on a monthly timestep. On the other hand, Chain 4 converges to a biologically unrealistic local optimum with a high probability of movement from Peoria directly into Dresden Island with very low movement probability among the pools in between (Scenario **B**)

With the Bayesian approach under uninformative priors, the MCMC chains optimized to two different solutions. In some chains, the detection probabilities reflected low movement and high detection probabilities, whereas others estimated high movement, low detection. These conflicting estimates occurred most notably in relation to detection and movement into the Dresden Island Pool, which has a very low number of observations (refer to Table 1). Compare the sets of transition probabilities estimated by separate chains in Fig. 5. For scenario A, strong movement occurs only between adjacent pools. For scenario B, movement is strong from Peoria directly into Dresden Island and very low among adjacent pools upriver of Peoria. Scenario B seems to be a very unlikely depiction of the true nature of this system given the effect of L & D structures on limiting fish movement in the river.

After biologically informative priors were included in the Bayesian approach and appropriate initial values were selected for the maximum likelihood estimation, both models produced similar parameter estimates. These constraints greatly improved the identifiability of the parameters and produced unique, biologically realistic estimates, and successful convergence was reported by a variety of statistics (refer to Section “Results”). Figure 4 gives a graphical depiction of these results, and these parameter estimates are also reported for direct comparison in the Additional file 1: Section 9.2.

Convergence issues due to weak identifiability may exist as a common issue among HMMs used for multi-state capture-recapture analyses. Due to these convergence challenges, modelers may want to consider whether parameter estimates correspond with prior knowledge of the biological system. Using informative priors for Bayesian methods and initial values for maximum likelihood methods may be used to guide model optimization toward biologically informed solutions when weak identifiability is an issue. Likewise, examining estimates carefully and questioning biologically inconsistent results can assist modelers in obtaining reliable results.

### Predicted spatial dynamics vary between estimation method

**Fig. 6:**
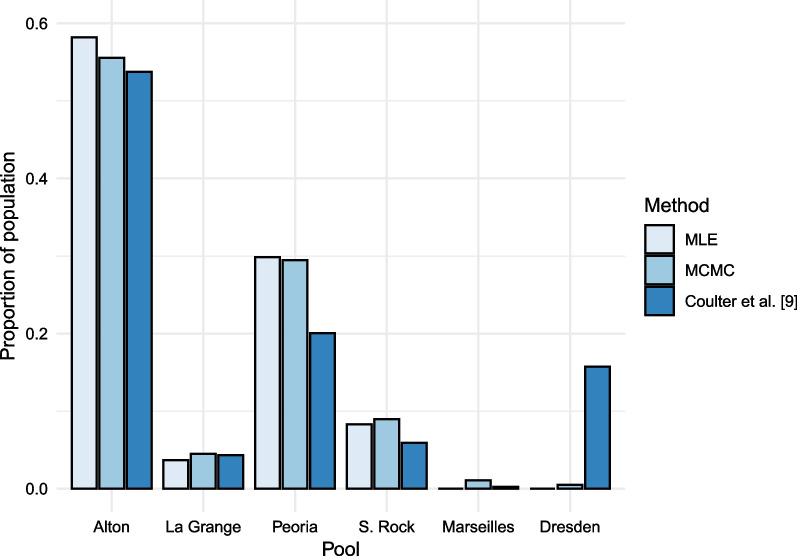
A comparison of the expected stable distributions based on movement alone for each method. Each bar represents the expected proportion of an initial silver carp population that would stabilize in each pool based on the maximum likelihood (MLE), Bayesian Markov-chain Monte Carlo (MCMC), and Coulter et al. [9] movement probability estimates. The totals of these bars for each method sum to one. There are notable differences in the expected stable distribution at the invasion front (Dresden Island) between these estimation methods, where no fish reach according to the MLE estimate but over 15% of all fish reach a stable state according to the previous Coulter et al. [9] results

Estimates of movement probabilities may be used to model population dynamics over time. We compared the expected stable distribution based on movement alone (i.e., ignoring births, deaths, and immigration/emigration outside of the system) using dominant eigenvectors of the system for each parameterization [39]. The distribution in Fig. 6 represents the expected proportion of the population that would eventually stabilize into each pool over many iterations.

These results indicate the estimation of movement probabilities strongly influences predicted population dynamics. Particularly in the Dresden Island Pool, differences in the predicted stable distribution are notable between models. The movement probabilities estimated in the previous work by Coulter et al. [9] predict over 15% of the silver carp population is expected to stabilize into this pool. Figure 4 shows the Coulter et al. [9] results indicate strong movement probability from Starved Rock into Dresden Island, but no movement from Starved Rock into Marseilles. This is a biologically unrealistic result, as all of the fish moving into Dresden Island from Starved Rock would first have to travel through Marseilles, which may be a sign the previous results suffer from weak identifiability. In contrast, our maximum likelihood results predict no silver carp will reach Marseilles or Dresden Island. The Bayesian MCMC approach estimates a small, but nonzero proportion of the population will stabilize in Marseilles and even fewer will reach Dresden Island. This result is most consistent with empirical studies of abundance in these pools [27].

Dresden Island is considered the current invasion front for silver carp in the Illinois River [16]. Using the results from the maximum likelihood analysis alone, a manager could conclude there is no risk of silver carp movement into the Dresden Island Pool. On the other hand, the previous Coulter et al. [9] results may indicate that reducing movement from downstream pools into Dresden Island by deterrents or removal would be beneficial. While the input data and model parameterization differed slightly between our HMM and the model presented in Coulter et al. [9], this comparison shows modeling decisions can have large implications on management needs and strategies.

### Tandem maximum likelihood and Bayesian MCMC estimation can improve model insight and credibility

We struggled to achieve convergence via both maximum likelihood and Bayesian MCMC estimation, but eventually produced similar estimates across both methods. The HMM is an appropriate model for multi-state capture-recapture movement data, but low frequency of observations in one or more HMM states can lead to poor convergence due to weak identifiability. Although the final maximum likelihood and Bayesian MCMC approaches return approximately equivalent estimates in terms of overall trends, a paucity of data still affects the maximum likelihood and Bayesian approaches in slightly different but influential manners. Given a lack of observations of movement between two pools, the maximum likelihood approach will likely return a movement probability equal to 0 between these pools, whereas the Bayesian MCMC approach will more likely return a small, but non-zero movement probability. This is an effect of the maximum likelihood optimization as a function of the data alone, whereas the Bayesian MCMC approach includes information from prior distributions.

We find that the implementation of tandem maximum likelihood and Bayesian analyses paired with a thorough examination of the results from both these methods can lead to a better understanding of the challenges and insights that may arise in any particular multi-state capture-recapture study. Similar convergence between both the maximum likelihood and Bayesian approaches increases the credibility of model results. When uninformative priors were used in the Bayesian MCMC approach there were not unique parameter estimates among the chains. Compared to the raw data, maximum likelihood estimates, and prior biological knowledge of the system, some of these parameter estimates were biologically unrealistic. Biologically informative priors ensured convergence across all MCMC chains and resulted in concordance between the maximum likelihood and Bayesian approaches.

In the end, however, the Bayesian approach handles the paucity of data among states with no observed movement in a slightly more realistic manner than the maximum likelihood approach. The Bayesian MCMC estimate reveals strong movement probabilities among adjacent pools and small, but nonzero probability that invasive carp move long distances over short time periods. Finally, we showed that incorporating these probabilities into a spatial prediction of population dynamics over time may lead to different management conclusions, especially when the maximum likelihood approach estimates no probability of movement into the invasion front and biologically unrealistic estimates of movement probability overestimate movement into the invasion front between distant pools.

We understand that some practitioners may find it difficult to fit both approaches due to a lack of previous experience and/or data limitations, but understanding the difficulties of fitting HMMs in general along with the underlying assumptions of either approach is critical to achieving reasonable parameter estimation. There is little evidence that either the maximum likelihood or Bayesian approach will always produce results that are more valid than the other. But a tandem implementation of both approaches can improve the credibility of parameter estimates when there is concordance between the results of both approaches. This is especially useful when a practitioner encounters difficulties in achieving convergence via either approach due to the identifiability challenges we have shown are present when fitting HMMs to multi-state capture-recapture studies with sparse data.

## Conclusions

HMMs represent multi-state capture-recapture data by modeling ecological phenomena, such as survival, movement, and imperfect detection. We described the fundamental nature of the HMM for both the maximum likelihood and Bayesian MCMC methods for fitting such models. However, our analysis of silver carp telemetry data in the Illinois River, along with the previous work by Coulter et al. [9], demonstrates several challenges that may be inherent in HMMs for multi-state capture-recapture studies. Computational methods for estimating model parameters in large models may have poor convergence when there are few observations of movement among one or more states due to weak parameter identifiability. Fitting these models may require constraining the model either through assumptions under a maximum likelihood framework or by using informative prior distributions in a Bayesian context.

We demonstrate these challenges may have a substantial effect on the conclusions and management recommendations drawn from these models. Implementing tandem maximum likelihood and MCMC approaches for fitting HMMs to multi-state capture-recapture data can improve model credibility when biologically realistic convergence patterns are achieved across approaches. However, we understand practitioners may find it difficult to fit both approaches due to a lack of previous experience and/or data limitations, but understanding the difficulties of fitting HMMs in general along with the underlying assumptions of either approach is critical to achieving reasonable parameter estimation.

This paper provides a foundation for scientists looking to implement multiple approaches for fitting HMMs to multi-state capture-recapture data. We discuss common challenges that may be encountered and the solutions that we found by studying silver carp telemetry data in the Illinois River. Investigating continuous time capture-recapture methods and neural network-based approaches may be beneficial for analyzing telemetry data [40, 41]. In this paper, we reduced the raw capture history for each individual to monthly time-steps, but continuous time models may be more compatible with capture-recapture data derived from acoustic telemetry arrays. Transitioning capture-recapture models from discrete time to continuous time models could further advance movement ecology as a big-data science [1].

### Supplementary Information

**Additional file 1.** The supplementary materials include additional details on the Bayesian MCMC priors and initial values specificied for the final model and a full report of final statistics for the maximum likelihood and MCMC parameter estimates.

## Data Availability

The raw silver carp telemetry dataset has been released separately by Alison Coulter under her affiliation with South Dakota State University. This release may be found at https://openprairie.sdstate.edu/nrm_datasets/3/ [42]. Non-authoritative copies of the data may also be found in the software release for this product and were included to more readily allow people to recreate our results. Code to reproduce the results of this paper may be found at https://doi.org/10.5066/P9JSNIIH [43].

## Abbreviations

HMM: Hidden Markov model
CJS: Cormack–Jolly–Seber
L & D: Lock & Dam
MCMC: Markov-chain Monte Carlo
rkm: River kilometer
MLE: Maximum likelihood estimate
