# Supplementary material for: Comparing maximum likelihood and Bayesian methods for fitting hidden Markov models to multi-state capture-recapture data of invasive carp in the Illinois River
Source: Mov Ecol. 2024 Jan 8;12:2. doi: 10.1186/s40462-023-00434-w (PMC10775585; doi:10.1186/s40462-023-00434-w)
Supplement: Supplementary file 1 — Additional file 1. The supplementary materials include additional details on the Bayesian MCMC priors and initial values specificied for the final model and a full report of final statistics for the maximum likelihood and MCMC parameter estimates. [file 40462_2023_434_MOESM1_ESM.pdf]

9 Supplementary Materials

9.1 Bayesian MCMC Priors and Initial Values

The survival and detection parameters were specified with the following priors:

$\phi \sim \text{Beta}(2,1)$ and $\rho_{k,t} \sim \text{Beta}(2,1)$ for $k \in 1,2,\ldots,6$ and $t \in 1,2,\ldots,4$.

The movement parameters $\psi_{j,k}$ for $j,k \in 1,2,\ldots,6$ were specified as transformations based on ratios of Gamma priors on the parameters $\xi_{j,k}$ for $j,k \in 1,2,\ldots,6$. For $j \in 1,2,\ldots,6$, let $\psi_{j,k} = \frac{\xi_{j,k}}{\sum_{k=1}^{6} \xi_{j,k}}$.

$\xi_{1,1} \sim \text{Gamma}(4,1)$	$\xi_{1,2} \sim \text{Gamma}(1,1)$	$\xi_{1,3} \sim \text{Gamma}(0.1,1)$	$\xi_{1,4} \sim \text{Gamma}(0.1,1)$	$\xi_{1,5} \sim \text{Gamma}(0.1,1)$	$\xi_{1,6} \sim \text{Gamma}(0.1,1)$
$\xi_{2,1} \sim \text{Gamma}(1,1)$	$\xi_{2,2} \sim \text{Gamma}(4,1)$	$\xi_{2,3} \sim \text{Gamma}(1,1)$	$\xi_{2,4} \sim \text{Gamma}(0.1,1)$	$\xi_{2,5} \sim \text{Gamma}(0.1,1)$	$\xi_{2,6} \sim \text{Gamma}(0.1,1)$
$\xi_{3,1} \sim \text{Gamma}(0.1,1)$	$\xi_{3,2} \sim \text{Gamma}(1,1)$	$\xi_{3,3} \sim \text{Gamma}(4,1)$	$\xi_{3,4} \sim \text{Gamma}(1,1)$	$\xi_{3,5} \sim \text{Gamma}(0.1,1)$	$\xi_{3,6} \sim \text{Gamma}(0.1,1)$
$\xi_{4,1} \sim \text{Gamma}(0.1,1)$	$\xi_{4,2} \sim \text{Gamma}(0.1,1)$	$\xi_{4,3} \sim \text{Gamma}(1,1)$	$\xi_{4,4} \sim \text{Gamma}(4,1)$	$\xi_{4,5} \sim \text{Gamma}(1,1)$	$\xi_{4,6} \sim \text{Gamma}(0.1,1)$
$\xi_{5,1} \sim \text{Gamma}(0.1,1)$	$\xi_{5,2} \sim \text{Gamma}(0.1,1)$	$\xi_{5,3} \sim \text{Gamma}(0.1,1)$	$\xi_{5,4} \sim \text{Gamma}(1,1)$	$\xi_{5,5} \sim \text{Gamma}(4,1)$	$\xi_{5,6} \sim \text{Gamma}(1,1)$
$\xi_{6,1} \sim \text{Gamma}(0.1,1)$	$\xi_{6,2} \sim \text{Gamma}(0.1,1)$	$\xi_{6,3} \sim \text{Gamma}(0.1,1)$	$\xi_{6,4} \sim \text{Gamma}(0.1,1)$	$\xi_{6,5} \sim \text{Gamma}(1,1)$	$\xi_{6,6} \sim \text{Gamma}(4,1)$

Initial values were selected randomly for ϕ and all $\rho_{k,t}$ where $k \in 1,2,\ldots,6$ and $t \in 1,2,\ldots,4$. The initial value for each $\xi_{j,k}$ where $j,k \in 1,2,\ldots,6$ was set to the mean of its specified Gamma prior.

9.2 Parameter Estimates

The first table reports estimates of the survival and detection parameters along with respective variance estimates for both the maximum likelihood (MLE) and Bayesian MCMC methods. The second table provides these estimates for the movement parameters. Note that any parameter with an `NA` value indicates the variance was not estimable due to the constraint structure and an `NaN` indicates a numerical approximation error (refer to Section 3 for more details).

	Pool	Season	MLE Estimate	MLE Standard Err.	MCMC Estimate	MCMC Standard Dev.	MCMC R-hat	MCMC ESS-Bulk	MCMC ESS-Tail
ϕ	All	All	0.8827	0.0068	0.8794	0.0062	1.0000	52975	29985
$\rho_{1,1}$	Alton	Dec-Feb	0.0668	0.0166	0.0757	0.0166	1.0001	60503	28630
$\rho_{1,2}$	Alton	Mar-May	0.1625	0.0313	0.1743	0.0277	1.0002	57624	30050
$\rho_{1,3}$	Alton	Jun-Aug	0.2389	0.0394	0.2533	0.0305	1.0000	55954	29196
$\rho_{1,4}$	Alton	Sep-Nov	0.0203	0.0103	0.0301	0.0122	1.0002	57299	24764
$\rho_{2,1}$	La Grange	Dec-Feb	0.7649	0.2124	0.5386	0.1637	1.0000	41737	17332
$\rho_{2,2}$	La Grange	Mar-May	1.0000	0.1839	0.9300	0.0617	1.0000	41216	19151
$\rho_{2,3}$	La Grange	Jun-Aug	0.8536	0.1478	0.8037	0.1243	1.0001	35020	18713
$\rho_{2,4}$	La Grange	Sep-Nov	0.8534	0.2980	0.7239	0.1733	1.0001	38528	20184
$\rho_{3,1}$	Peoria	Dec-Feb	0.1389	0.0205	0.1479	0.0222	1.0002	58890	28083
$\rho_{3,2}$	Peoria	Mar-May	0.2100	0.0260	0.2237	0.0294	1.0000	56847	29034
$\rho_{3,3}$	Peoria	Jun-Aug	0.1180	0.0194	0.1281	0.0215	1.0003	56514	29270
$\rho_{3,4}$	Peoria	Sep-Nov	0.0367	0.0149	0.0515	0.0182	1.0001	59203	27365
$\rho_{4,1}$	Starved Rock	Dec-Feb	0.9092	0.0273	0.9028	0.0272	1.0003	57034	24482
$\rho_{4,2}$	Starved Rock	Mar-May	0.9490	0.0286	0.9374	0.0295	1.0001	41669	18671
$\rho_{4,3}$	Starved Rock	Jun-Aug	0.8631	0.0467	0.8529	0.0455	1.0000	55217	24228
$\rho_{4,4}$	Starved Rock	Sep-Nov	0.9542	0.0259	0.9447	0.0261	1.0000	48750	20202
$\rho_{5,1}$	Marseilles	Dec-Feb	0.8309	0.0604	0.8074	0.0467	1.0001	40007	23639
$\rho_{5,2}$	Marseilles	Mar-May	0.9875	0.0323	0.9598	0.0282	1.0001	29890	15707
$\rho_{5,3}$	Marseilles	Jun-Aug	0.7059	0.0743	0.7004	0.0653	1.0000	45202	24586
$\rho_{5,4}$	Marseilles	Sep-Nov	0.8087	0.0756	0.7911	0.0577	1.0001	43461	22858
$\rho_{6,1}$	Dresden Island	Dec-Feb	0.0000	0.2519	0.0416	0.0299	1.0000	48327	22829
$\rho_{6,2}$	Dresden Island	Mar-May	0.0478	0.0342	0.1026	0.0506	1.0001	55463	25883
$\rho_{6,3}$	Dresden Island	Jun-Aug	0.1775	0.0700	0.2549	0.0902	1.0001	47733	27948
$\rho_{6,4}$	Dresden Island	Sep-Nov	0.0000	0.5263	0.0957	0.0701	1.0001	50851	23550

	From	To	MLE Estimate	MLE Standard Err.	MCMC Estimate	MCMC Standard Dev.	MCMC R-hat	MCMC ESS-Bulk	MCMC ESS-Tail
$\psi_{1,1}$	Alton	Alton	0.9467	0.0253	0.9386	0.0115	1.0001	41767	33266
$\psi_{1,2}$	Alton	La Grange	0.0399	0.0078	0.0479	0.0099	1.0000	42443	31922
$\psi_{1,3}$	Alton	Peoria	0.0133	0.0061	0.0130	0.0055	1.0000	54603	27892
$\psi_{1,4}$	Alton	Starved Rock	0.0000	0.0026	0.0002	0.0007	1.0001	16135	15239
$\psi_{1,5}$	Alton	Marseilles	0.0000	0.0168	0.0001	0.0004	1.0001	17227	15660
$\psi_{1,6}$	Alton	Dresden Island	0.0000	0.0212	0.0002	0.0005	1.0001	16339	15461
$\psi_{2,1}$	La Grange	Alton	0.7161	0.1212	0.6571	0.0525	1.0000	45667	32616
$\psi_{2,2}$	La Grange	La Grange	0.2315	0.0540	0.2767	0.0475	1.0000	43849	30370
$\psi_{2,3}$	La Grange	Peoria	0.0431	0.0276	0.0552	0.0283	1.0001	55306	25336
$\psi_{2,4}$	La Grange	Starved Rock	0.0092	0.0091	0.0092	0.0087	1.0000	44024	18925
$\psi_{2,5}$	La Grange	Marseilles	0.0000	0.2341	0.0008	0.0024	1.0008	16862	14788
$\psi_{2,6}$	La Grange	Dresden Island	0.0000	0.2889	0.0010	0.0032	1.0002	16918	15926
$\psi_{3,1}$	Peoria	Alton	0.0161	0.0074	0.0156	0.0066	1.0000	58847	29211
$\psi_{3,2}$	Peoria	La Grange	0.0159	0.0045	0.0191	0.0053	1.0001	45286	31974
$\psi_{3,3}$	Peoria	Peoria	0.9188	0.0331	0.9136	0.0120	1.0000	40590	30879
$\psi_{3,4}$	Peoria	Starved Rock	0.0493	0.0084	0.0514	0.0085	1.0000	42445	30687
$\psi_{3,5}$	Peoria	Marseilles	0.0000	0.0288	0.0001	0.0004	1.0001	16786	15065
$\psi_{3,6}$	Peoria	Dresden Island	0.0000	0.0539	0.0002	0.0005	1.0001	16695	15122
$\psi_{4,1}$	Starved Rock	Alton	0.0000	0.0025	0.0005	0.0013	1.0000	15236	13424
$\psi_{4,2}$	Starved Rock	La Grange	0.0017	0.0001	0.0018	0.0017	1.0003	43764	20768
$\psi_{4,3}$	Starved Rock	Peoria	0.1802	0.0212	0.1748	0.0175	1.0001	40302	27794
$\psi_{4,4}$	Starved Rock	Starved Rock	0.8181	0.0402	0.8213	0.0176	1.0001	39868	28320
$\psi_{4,5}$	Starved Rock	Marseilles	0.0000	0.0625	0.0014	0.0015	1.0000	43278	20683
$\psi_{4,6}$	Starved Rock	Dresden Island	0.0000	0.0544	0.0002	0.0006	1.0002	15949	14973
$\psi_{5,1}$	Marseilles	Alton	0.0000	0.0615	0.0004	0.0013	1.0001	16482	15868
$\psi_{5,2}$	Marseilles	La Grange	0.0000	0.0551	0.0002	0.0007	1.0005	16314	15155
$\psi_{5,3}$	Marseilles	Peoria	0.0093	0.0084	0.0087	0.0063	1.0000	42977	19974
$\psi_{5,4}$	Marseilles	Starved Rock	0.0301	0.0081	0.0316	0.0080	1.0001	44691	31018
$\psi_{5,5}$	Marseilles	Marseilles	0.8425	0.0645	0.8624	0.0271	1.0002	30601	30834
$\psi_{5,6}$	Marseilles	Dresden Island	0.1182	0.0300	0.0966	0.0254	1.0001	31028	31984
$\psi_{6,1}$	Dresden Island	Alton	0.0000	0.0346	0.0014	0.0044	1.0001	15377	12926
$\psi_{6,2}$	Dresden Island	La Grange	0.0000	0.0416	0.0009	0.0030	1.0001	16569	13888
$\psi_{6,3}$	Dresden Island	Peoria	0.0000	0.0256	0.0025	0.0069	1.0000	15029	14251
$\psi_{6,4}$	Dresden Island	Starved Rock	0.0000	0.0175	0.0011	0.0035	1.0002	15898	14648
$\psi_{6,5}$	Dresden Island	Marseilles	0.2382	0.0651	0.2459	0.0548	1.0001	42431	29957
$\psi_{6,6}$	Dresden Island	Dresden Island	0.7618	0.0640	0.7482	0.0553	1.0001	42200	30087
